# In vitro study of the antioxidative and antiproliferative capabilities of *Lactobacillus casei* 16‐fermented soymilk

**DOI:** 10.1002/fsn3.1214

**Published:** 2019-12-11

**Authors:** Fang Qian, Jiang Zhang, Kairong Hou, Yuqing Zhang, Zifei Wang, Pengjie Luo, Yanfeng Tuo

**Affiliations:** ^1^ School of food science and technology Dalian Polytechnic University Dalian China; ^2^ NHC Key Laboratory of Food Safety Risk Assessment China National Center for Food Safety Risk Assessment Beijing China

**Keywords:** antioxidation, antiproliferation, fermentation, soymilk

## Abstract

In this study, soymilk was fermented with *Lactobacillus casei* 16. The contents of aglycone isoflavones, saponins, total phenolic, and free amino acid in the fermented soymilk, and the protection for the HepG_2_ cells against 2,2'‐azobis(2‐amidinopropane) dihydrochloride (ABAP) damage and the antiproliferative effects of the fermented soymilk on the HT‐29 cells and Caco‐2 cells were studied. The results showed that the levels of total phenolic, aglycone isoflavones, and free amino acids in the *L. casei* 16‐fermented soymilk were enhanced. The ethanol extract and the water extract of the *L. casei* 16‐fermented soymilk showed protection for HepG_2_ cells against ABAP damage and inhibited the proliferation of the HT‐29 cells and Caco‐2 cells, which may be attributed to the enhanced level of total phenolic, aglycone isoflavones, and free amino acids in the *L. casei* 16‐fermented soymilk.

## Graphical abstract

Soymilk fermented with *Lactobacillus casei* 16 showed the enhanced levels of total phenolic, aglycone isoflavones, and free amino acids. *L. casei* 16 fermented soymilk showed protection for HepG_2_ cells against ABAP damage and inhibited the proliferation of human colon cancer cells, which may be attributed to the enhanced level of total phenolic, aglycone isoflavones, and free amino acids in the *L. casei* 16‐fermented soymilk.

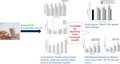

## 1 INTRODUCTION

Soybean is rich in polyphenol, protein, and fatty acids and consumed in the forms of soymilk, tofu, soy sauce, tempeh, and so on (Dajanta & Chukeatirote, 2012; Erdman & Committee, 2000). Soymilk is manufactured by grounding soybean with water in some ratio. Soymilk was greeting by some consumers due to its protein, fatty acid, and isoflavones. On the other hand, soymilk was disgusting due to the undesirable bean odor and the flatulence.

Soymilk fermentation by some microbes including lactic acid bacteria can enhance the nutritional availability and physiological functions of the soymilk due to the bioconversion of some complex organic compounds into bioactive compounds (Sanjukta & Rai, 2016). Several studies have demonstrated that the fermentation by some kind of microorganism could increase the aglycone isoflavone (Donkor & Shah, 2010; Jane, Monique, FrançOise, FrançOis, & Jean, 2008; Jiyeon et al., 2010; Marazza, Garro, & de Giori, 2009), total phenolic content (Han, Hur, & Lee, 2015; Landete, Curiel, Rodríguez, Rivas, & Muñoz, 2014), and free amino acid content (Chen, Chiang, Chou, & Lo, 2013; Sanjukta, Rai, Muhammed, Jeyaram, & Talukdar, 2015; Zhang, Tatsumi, Fan, & Li, 2010) and reduce the content of saponins (Lai, Hsieh, Huang, & Chou, 2013; Rui et al., 2017). These microbes could hydrolyze the glucose moiety of isoflavones conjugates due to β‐glucosidase and promote biological activity of soybean products (Pham & Shah, 2008; Wei, Chen, & Chen, 2007; Zhai et al., 2014). The proteins in soybean products could be hydrolyzed into peptide or free amino acid duo to the protease of the starter cultures (Guan et al., 2017).

The nutritional content changes of the fermented soybean and their products could account for some enhanced physiological functions of the fermented soybean and their products. In general, antioxidative activities of the fermented soybean products are significantly higher than those of their nonfermented counterpart (Marazza, Nazareno, Giori, & Garro, 2012; Yang et al., 2017). Fermented soybean products exhibited higher 1,1‐diphenyl‐2‐picrylhydrazyl (DPPH) radical scavenging activity, 2,2'‐Azinobis‐(3‐ethylbenzthiazoline‐6‐sulfonate (ABTS) radical scavenging activity, ferric reducing antioxidant power (FRAP) reducing power, hydroxyl radical scavenging activity, and superoxide radical scavenging activity than those of the unfermented soybean products (Lee, Hung, & Chou, 2008; Ming‐Yen & Cheng‐Chun, 2010; Moktan, Saha, & Sarkar, 2008). Dajanta, Janpum, and Leksing. (2013) reported that the FRAP values were correlated well with the enhanced total phenolic contents in *Bacillus subtilis* TN51‐fermented soybeans. Hu et al. (2010) reported that the values of scavenging activity toward DPPH radicals in black soybeans fermented by *Bacillus natto* showed positive correlation with the enhanced contents of total phenols and aglycone isoflavone.

The food with antioxidant capacities was reported to exert anticancer activities. Wen‐Huei, Jun‐Jen, Ching‐Hsein, Tien‐Shang, and Fung‐Jou. (2002) demonstrated that fermented soymilk product (FSP) has a growth‐inhibitory effect on various human breast carcinoma cell lines, especially on MCF‐7 cells. The soymilk fermented with *Bifidobacterium breve* Yakult could inhibit female Sprague Dawley rats mammary carcinogenesis due to the increased content of isoflavone aglycone (Ohta et al., 2000).

In our previous work, *Lactobacillus casei* 16 showed higher β‐glucosidase activity, and the *L. casei* 16‐fermented soymilk exhibited the DPPH free radical scavenging capacity and oxygen radical absorbance capacity (ORAC) (Tang et al., 2018). In this study, we further investigated the effect of fermentation by *L. casei* 16 on the aglycone isoflavone, total phenolic, free amino acid, and saponin content in the fermented soymilk. In addition, the ethanol and water extracts of the fermented soymilk were studied for the protection for HepG_2_ cell against ABAP damage and the antiproliferative effect on human colon cancer cell lines.

## 2 MATERIALS AND METHODS

### 2.1 Propagation of cultures

The strain *Lactobacillus casei* 16 was provided by Dalian probiotic functional property key laboratory in Dalian Polytechnical University. *L. casei* 16 was stored in MRS broth containing 25% glycerol at − 80◦C. *L. casei* 16 was grown in 5 ml sterile MRS medium and incubated at 37◦C for 18 hr. After 2 successive culture in MRS medium, 5 ml aliquots of sterile soymilk were incubated (2% v/v) with *L. casei 16* previously activated.

### 2.2 Preparation of soymilk and soymilk fermentation

The soymilk preparation and fermentation by *L. casei* 16 was carried out according to our previous work (Tang et al., 2018). Soybean seeds were obtained from a local supermarket and stored at 4◦C until use. Whole soybeans were cleaned by washing and soaked overnight in 2 times their weight of distilled water. The soaked soybeans were blended with 4 times their weight of distilled water and ground in a blender for 3 min. And the ground mixture was filtered through a double‐layered cloth to obtain soymilk. The soymilk was dispensed into sterile bottle and sterilized by autoclaving for 15 min at 105°C. After cooling, the sterile soymilk was inoculated with *L. casei* 16 by 2% (v/v) and incubated at 37 ◦C for 24 hr.

### 2.3 Preparation of solvent extracts

The method of the extraction was according to our previous work (Tang et al., 2018). To prepare solvent extracts, nonfermented or fermented soymilk was freeze‐dried using freeze dryer (FD‐IC‐5D, Bo Medical Experimental Instrument Co., Ltd.), and the freeze‐dried soymilk or fermented soymilk was extracted by ultrasound (100 W) with solvent (water or 80% ethanol) (1:10, w/v) and held at 25°C for 6 hr. The extracts were centrifuged at 10,000 *g* for 10 min at 4°C before the supernatants were collected. The supernatants were freeze‐dried again. The freeze‐dried extracts were stored at −80°C.

### 2.4 Determination of total phenolic content

The total phenolic contents of samples were examined as described by Chen et al. (2013) with minor modifications. The freeze‐dried samples were dissolved in 0.1 ml DMSO (Merck).The 1.9 ml of deionized water and 1.0 ml of Folin–Ciocalteu phenol reagent (Sigma‐Aldrich Co.) were added to the solution. Then, 5.0 ml of 20% Na_2_CO_3_ was added to the mixture at room temperature in darkness and allowed to react for 20 min. Absorbance of samples was then measured at 735 nm. The total phenolic content of the sample was compared with a standard curve of prepared gallic acid solution, and the results were expressed as milligrams of gallic acid per milligram of extract.

### 2.5 The free amino acid content measurement

The free amino acid contents of the fermented and unfermented soymilk were measured as described by Xing et al. (2017) with minor modifications. The pH of unfermented and fermented soymilk was adjusted to 4.6 before determination. The samples were then centrifuged at 3,000 *g* for 30 min at 4°C to obtain supernatants. Fifty microliters of the supernatants was mixed with 2 ml O‐phthalaldehyde and incubated for 2 min at room temperature. The absorbance of samples was read at 340 nm. The free amino acid content was determined from a standard curve constructed with Leucine standard, and the results were expressed as milligrams of Leucine extract.

### 2.6 Quantification of total saponin

The saponin quantification in the soymilk was determined as described by Helaly method (Dini, Schettino, Simioli, & Dini, 2001) with slight modification. Briefly, the freeze‐dried samples were dissolved in MeOH 80%. Aliquots of the samples (0.1 ml) were mixed with fresh made vanillin solution (8% in ethanol, 0.1 ml) and added to sulfuric acid (72%, 1 ml). The mixtures were allowed to bath at 60°C water for 20 min and then stand for 5 min at ice‐cold water. The absorbance of the mixture at 544 nm was measured, and saponin content was calculated from a standard curve constructed with purified soyasaponin standard.

### 2.7 HPLC analysis of the soy isoflavone

The soy isoflavone contents in the soymilk were determined according to our previous work (Tang et al., 2018). The ethanol extracts of soymilk and fermented soymilk were redissolved in 80% methanol. The samples were filtered through a 0.22 μm‐pore‐size polyvinylidene fluoride filter (PVDF) (Teknokroma, Barcelona, Spain) prior to analyzed by HPLC. The analytical HPLC was consisted with Waters 2,695 Alliance, a Waters 2,998 PDA detector, and C18 column (Optimapak, 4.5 × 250 mm, 5 μm). The autoinjector was used to inject 20 μl of the samples, and isoflavone was detected by monitoring the elution at 260 nm. The identification of isoflavones was calculated from retention time, and PDA spectrums were compared with those of the standards (genistein, genistin, daidzin, and daidzein, purchased from Sigma‐Aldrich chemical company). The mobile phase was composed of 0.1% acetic acid in distilled water (solution A) and acetonitrile (solution B), and the solvent flow rate was maintained at 1 ml/min. The gradient conditions were as follows: 0–28 min, 85% A, 1.0 ml/min; 28–42 min, 65% A, 1.0 ml/min; 42–47 min, 55% A, 1.0 ml/min; and 47– 59 min, 85% A, 1.0 ml/min.

### 2.8 Cell culture

The human colon cancer cell lines HT‐29 and Caco‐2, and liver cancer cell line HepG_2_ used in this study were purchased from the Cell Bank of the Type Culture Collection of the Chinese Academy of Sciences, in Shanghai, China. HT‐29, Caco‐2, and HepG_2_ cells were routinely cultured at 37°C in a 5% CO_2_ and 95% air atmosphere. HT‐29 cells were grown in Roswell Park Memorial Institute (RPMI) 1,640 medium (Gibco Life Technologies) supplemented with 10% (v/v) heat‐inactivated fetal bovine serum (Gibco Life Technologies), penicillin (100 U/ml), and streptomycin (100 U/ml; Sigma‐Aldrich). HepG_2_ and Caco‐2 cells were grown in DMEM medium (Gibco Life Technologies) supplemented with 20% (v/v) heat‐inactivated fetal bovine serum (Gibco Life Technologies), penicillin, and streptomycin (100 U/ml; Sigma‐Aldrich).

### 2.9 Cytotoxicity

Cytotoxicity was measured using the method of Wolfe and Hai (2008) with modifications. 100 μl of HepG_2_, Caco‐2, and HT‐29 cells at a concentration of 4 × 10^5^ Cell/ml was seeded in the wells of 96‐well plate. After incubation for 24 hr at 37°C, the grown medium was taken out, and the wells were washed with PBS twice. Then, the wells of experimental group were added with soymilk or fermented soymilk at the concentration of 50 μg/ml, 125 μg/ml, 250 μg/ml, and 500 μg/ml, respectively, and the plates were incubated at 37°C for 24 hr. After the medium was removed, the wells were washed with PBS twice, and the plate was added with fifty microliter methylene blue staining solution (98% HBSS, 0.67% glutaraldehyde and 0.6% methylene blue). After incubation at 37°C for 1 hr, the methylene blue was removed; the plate was washed with fresh distilled water several times and then the plate dried in room temperature. 100 μl of elution solution (49% PBS, 50% ethanol and 1% acetic acid) was added to the dried plate and incubated on a bench‐top shaker for 20 min at room temperature. The absorbance of the wells in the plate was determined at 570 nm using Multiskan GO microplate reader (Thermo Fisher Scientific). The absorbance decreased by >10%, compared with the control, was considered to be cytotoxic.

### 2.10 Effects of soymilk extracts on Survival of HepG_2_ cells by ABAP oxidative damage

The ABAP oxidative damage was measured using the method of Song et al. (2010) with some modifications. HepG_2_ cells were seeded in the wells in a 96‐well plate at a density of 1 × 10^5^ cell/ml using 100 μl of growth medium and incubated for 24 hr at 37°C to allow for cell attachment. After 24 hr incubation, the growth medium was removed and washed with PBS twice. The cells were treated with 100 µl ABAP (final concentration of 50 mmol/L, 60 mmol/L, 70 mmol/L, 75 mmol/L, 80 mmol/L, 85 mmol/L, 90 mmol/L, and 100 mmol/L dissolved in DEME medium, respectively, Sigma‐Aldrich）or DEME medium of 0 mmol/L ABAP as control for up to 2 hr at 37°C. To assess cell viability, the modified methylene blue assay was to be used. The viability of HepG_2_ cells was measured at 570 nm using Multiskan GO microplate reader (Thermo Fisher Scientific). The semilethal concentration (IC50) was determined. The HepG_2_ cell survival rate was calculated using the following equation:

$$\text{Survival}\;\;\text{rate}\;\;\left(\%\right)=\left[\left(A_{\text{sample}}{\;\text{- A}}_{\text{blank}}\right)/\left(A_{\text{control}}{\;\text{- A}}_{\text{blank}}\right)\right]/\text{Acontrol}\times 100.$$

where A_control_ is the absorbance at 570 nm of the control, A_sample_ is the absorbance at 570 nm of the sample, and A_blank_ is the optical absorbance at 570 nm of the blank group. All samples were analyzed in triplicate.

To evaluate the protective effect of dried samples on HepG_2_ cells against ABAP injury, HepG_2_ cells were seeded at a density of 1 × 10^5^ Cell/ml on a 96‐well plate in 100 μl of growth medium and incubated for 24 hr at 37°C. After cells adhere to the wall, the experimental group was fed with soymilk and fermented soymilk at the final concentration of 125 μg/ml, 250 μg/ml, and 500 μg/ml for one hour. Cells were treated with ABAP at the concentration of IC50 to induce oxidative stress for 2 hr and fresh medium as control. The HepG_2_ cell viability was calculated as described above.

### 2.11 Effects of soymilk extracts on Superoxide Dismutase (SOD) in HepG_2_ cells by ABAP oxidative damage

To evaluate the effects of fermented soymilk extracts on the production of SOD in HepG_2_ cells by ABAP oxidative damage, HepG_2_ cells were cultured in 96‐well plate as described above. Then, the HepG_2_ cells were treated with different extracts at the final concentration of 125 μg/ml, 250 μg/ml, and 500 μg/ml for one hour. Cells were treated with ABAP at the concentration of IC50 to induce oxidative stress for 2 hr and fresh medium as control. Cultured medium was removed, and cells were then lysed in a suitable volume of buffer (50 mM Tris–HCl pH 8.0, 50 mM EDTANa2, 0.2 M NaCl, 1% Triton X‐100). Cell homogenate was then centrifuged at 10,000 × g, 4°C for 10 min at 4°C. The afforded supernatant was stored at −20°C prior to the assays. Superoxide dismutase (SOD) activity was determined using the detection kit provided by Nanjing Jiancheng Bioengineering Institute (Nanjing, China).

### 2.12 Effects of fermented soymilk extracts on human colon cancer cells proliferation

After the assessing of the fermented and unfermented soymilk extracts cytotoxicity, the antiproliferative activity of the crude extracts was measured using methylene blue assay as described above. HT‐29 cells and Caco‐2 cells were seeded in the wells of 96‐well plates at a density of 5 × 10^4^ cells/ml in 200 μl of fresh medium for 24 hr. Cells were then treated with the extracts at different concentration (50μg/ml, 125 μg/ml, 250 μg/ml, 500 μg/ml) and incubated for 72 hr at 37°C. The inhibition rates of HT‐29 cells and Caco‐2 cells were calculated using the following equation:

$$\text{Inhibition}\;\;\text{rate}\;\;\left(\%\right)=\left[1-\left(\text{absorbance}\;\;\text{in}\;\;\text{test}\;\;\text{well}\right)/\left(\text{absorbance}\;\;\text{in}\;\;\text{control}\;\;\text{well}\right)\right]\times 100\%.$$

### 2.13 Western Blot Assay

The Western blot analysis was carried out as Mu et al. (2018) with some modifications. All antibodies were purchased from Beyotime Institute of Biotechnology. HT‐29 cells were added into wells of 6‐well plates (Corning Inc.) at a density of 1.0 × 10^6^ cells/ml for 48 hr. Then, cells were pretreated with 2 ml sample (the water extract of fermented and nonfermented soymilk dissolved in RPMI1640 medium) for 48 hr. The medium was removed and washed thrice with ice‐cold sterile PBS. Cells scraped from the wells were suspended in radio‐immunoprecipitation assay (RIPA) buffer (Solarbio life science) with 1% phenylmethanesulfonyl fluoride (PMSF, Solarbio life science). Cell lysates were held at 4°C with gentle ultrasound to facilitate protein extraction. Lysates was immediately centrifuged (10,000 × *g*, 4°C for 10 min). Protein concentrations of the lysates were determined using bicinchoninic acid (BCA) protein assay kit (Solarbio life science). Equal amounts of protein from each cell lysate were loaded onto 12% (w/v) sodium dodecyl sulfate–polyacrylamide gel electrophoresis (SDS‐PAGE) and transferred to polyvinylidene fluoride (PVDF) membranes. The membranes were treated with PBST (PBS containing of 0.05% Tween 20) containing 5% nonfat milk to blocked for 1 hr at room temperature. Membranes were incubated with antibodies PCNA and β‐actin overnight at 4°C. After the membranes were washed with PBST, the secondary antibodies were incubated for 1 hr at 4°C. The protein combine with antibodies was visualized by BeyoECL star Kit (Beyotime Institute of Biotechnology). Bands were then scanned and quantified by ImageJ software (National Institutes of Health, Bethesda, USA). The expression of human β‐actin was used as an internal standard control. All measurements were conducted in triplicate.

### 2.14 Statistics

All the experiments were repeated 3 times or more, the results were expressed as mean standard deviation, and the results were statistically analyzed by SPSS 20.0 software. The multiple comparisons were tested by Duncan's test, and the difference was significant ( *p* < .05).

## 3 RESULTS AND DISCUSSION

The strain *L. casei* 16 was screened in our previous work (Tang et al., 2018). *L. casei* 16 had higher β‐glucosidase activity, and the *L. casei* 16‐fermented soymilk showed antioxidant activities evidenced by the DPPH free radical scavenging capacity and ORAC value (Tang et al., 2018). In this study, the contents of total phenolic, aglycone isoflavones, and free amino acid in the *L. casei* 16‐fermented soymilk were determined, and the cellular antioxidant activity and the antiproliferative effects on human colon cancer cell lines of the fermented soymilk were studied.

### 3.1 The total phenolic, aglycone isoflavones, free amino acids, and saponin levels in the soymilk fermented by *L. casei* 16

**Figure 1:**
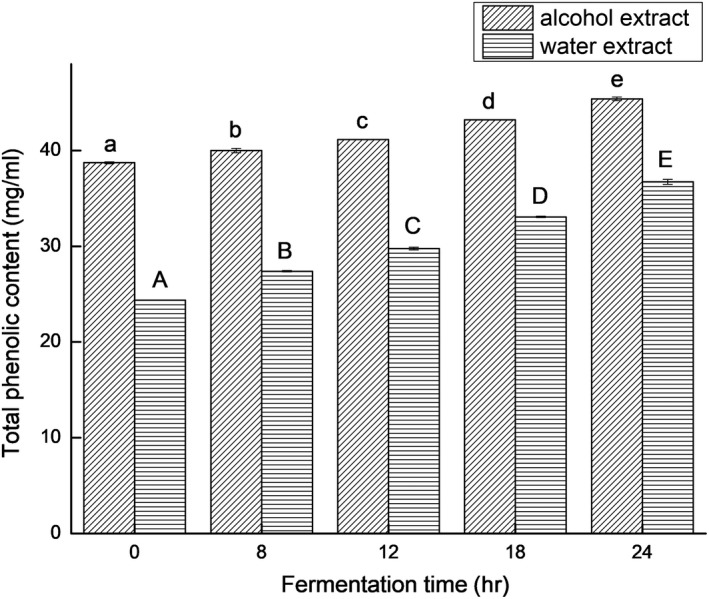
The total phenolic contents of soymilk fermented by *Lactobacillus casei* 16 at 37°C for different time. Data are represented as mean ± *SD* (*n* = 3). Mean values in the same group with different letters (a‐e; A‐E) are significantly different by Duncan's multiple range test (*p* < .05)

**Figure 2:**
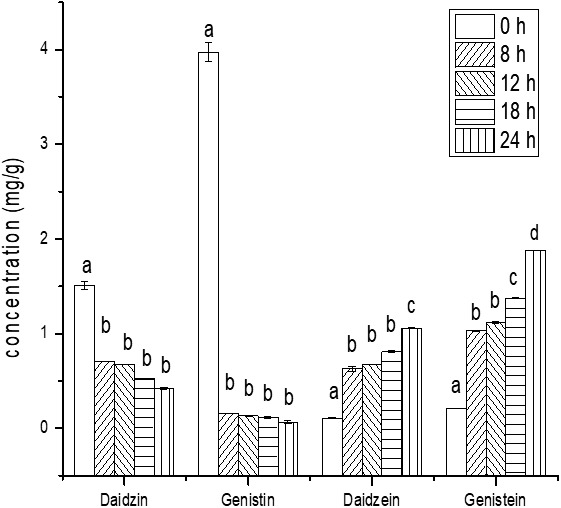
The concentration of the soybean isoflavone in the soymilk and the fermented soymilk (mg/g dry soymilk). Data are represented as mean ± *SD* (*n* = 3). Mean values in the same group with different letters (a, b, c, d) are significantly different by *t* test (*p* < .05)

**Figure 3:**
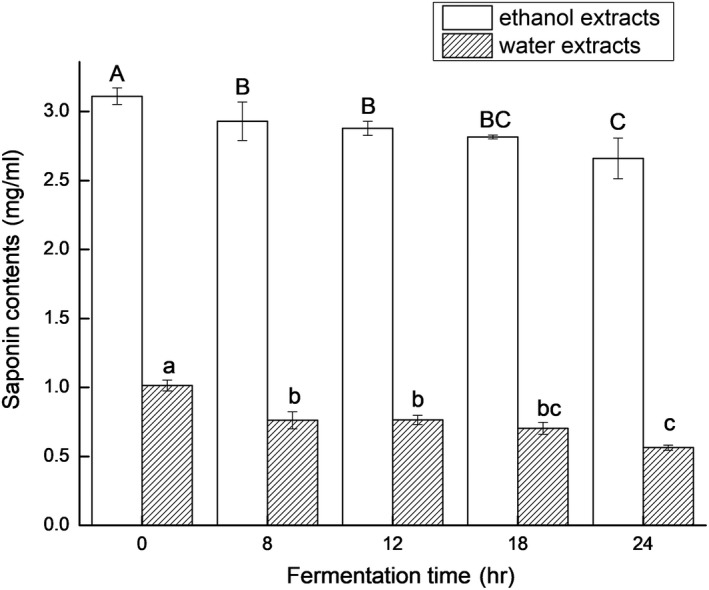
The saponin content of soymilk fermented by *Lactobacillus casei* 16 at 37°C for different time. Data are represented as mean ± *SD* (*n* = 3). Mean values in the same group with different letters (a‐c; A‐C) are significantly different by Duncan's multiple range test (*p* < .05)

In this study, the total phenolic content of the soymilk fermented by *L. casei* 16 significantly increased (*p* < .05) with the prolonging of fermentation time, as shown in Figure 1. The aglycone isoflavones, daidzein and genistein, concentrations in the fermented soymilk significantly increased with fermentation time prolonging (*p* < .05), while the glucosides isoflavones concentrations in the fermented soymilk decreased significantly, as shown in Figure 2. The increase in aglycone isoflavones could be due to the bioconversion of glucosides isoflavones by *L. casei* 16. As shown in Figure 3, with the prolonging of fermentation time, the saponin contents of the ethanol extracts and water extracts of the *L. casei* 16‐fermented soymilk reduced. These consequences might be caused by increased β‐glucosidase activity in the *L. casei* 16‐fermented soymilk. The recovery of isoflavones and saponins was 91.81% and 94.40%.

**Figure 4:**
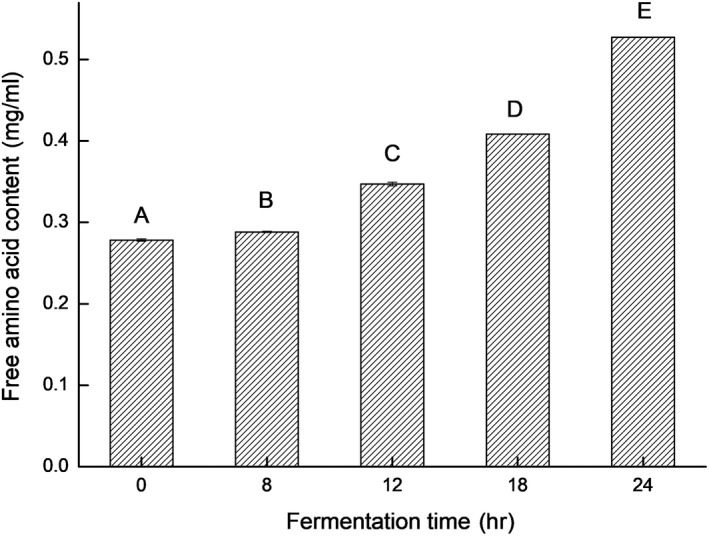
The free amino acid content of soymilk fermented by *Lactobacillus casei* 16 at 37°C for different time. Data are represented as mean ± *SD* (*n* = 3). Mean values in the same group with different letters (A‐E) are significantly different by Duncan's multiple range test (*p* < .05)

The free amino acid contents of the *L. casei* 16‐fermented soymilk increased significantly (*p* < .05) with prolonging fermentation time, as shown in Figure 4. A higher amino nitrogen content implied a higher degree of protein hydrolysis and higher contents of amino acid and peptides in the sample.

Our previous work found that the β‐glucosidase activities of the *L. casei* 16‐fermented soymilk increased significantly during the fermentation time ranging from 6 to 24 hr (Tang et al., 2018). Catalyzing the release of phenolics by β‐glucosidase of *L. casei* 16 during fermentation could account for the increase in the total phenolic content and aglycone isoflavones contents. Rekha and Vijayalakshmi (2011) reported that the isoflavone glucosides in soymilk could be converted to aglycone isoflavone catalyzed by β‐glucosidase produced by some lactobacilli strains. The β‐glucosidase produced by lactic acid bacteria is responsible for the breakdown of β‐1–6 glucosidic bond, which conjugates the pran ring of isoflavone and the sugar moieties (Donkor & Shah, 2010). The breakdown of isoflavone glycosides into sugar moieties and bioactive isoflavone aglycones during fermentation could improve the biological activity of soymilk. The β‐glucosidase catalytic action of *L. casei* 16 could lead to the reduction of saponin content in the fermented soymilk too. β‐glucosidase can split sugar side chains of steroid and triterpenoid saponins and lower the water solubility of the compounds. Yan et al. (2018) found that the β‐glucosidase BIBG3 from *B. longum* catalyzed the hydrolysis of saponin at higher efficiency. Chen et al. (2013) reported that the peptide and amino acid contents of black soybeans fermented by *Aspergillus awamori* were enhanced. Su, Cheng, Hsiao, Han and Yu (2018) reported that the solid‐state fermentation of soybean by *Lactobacillus* species and Clostridium butyricum could increase the degradation of soybean protein. Watanabe, Fujimoto, and Aoki (2007) suggested that antioxidant activity in the water‐soluble fraction of Rhizopus‐fermented tempeh may be due to the amino acids and peptides formed during fermentation. In this study, the fermentation of soymilk by *L. casei* 16 also increased the free amino acid contents, which may be ascribed to the proteases produced by *L. casei* 16.

### 3.2 Effects of fermented soymilk extracts on the survival of HepG_2_ cells treated by ABAP

In this study, whether the fermented soymilk ethanol extracts or water extract protect HepG_2_ cells against ABAP damage was studied. ABAP is a kind of oxidant and can damage normal cells. The ABAP‐damaged HepG_2_ cell model was established in this study. The survival rates of the ABAP‐damaged HepG_2_ cells decreased with the increase in ABAP dosage. When the ABAP concentration was 85 mmol/L, the HepG_2_ cell survival rate was close to 50%. So the semilethal concentration of ABAP to HepG_2_ cells was 85 mmol/L (IC50).

**Figure 5:**
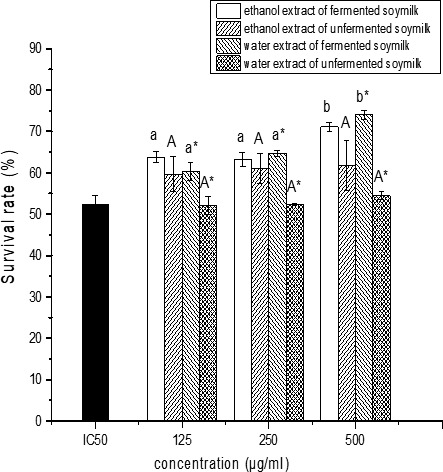
The effect of the extracts of the fermented and unfermented soymilk at different concentrations on the survival rate (%) of the HepG_2_ cells damaged by ABAP. Data are represented as mean ± *SD* (*n* = 3). Mean values in the same group with different letters (a‐c; A‐C; a*‐b*; A*) are significantly different by Duncan's multiple range test (*p* < .05)

And the water or ethanol extracts of *L. casei* 16‐fermented or *L. casei* 16‐unfermented soymilk at the concentrations ranging from 50 μg/ml to 500 μg/ml had no cytotoxic effects on HepG_2_ cells. While the ethanol extract and water extract of *L. casei* 16‐fermented soymilk could enhance the survival rates of the HepG_2_ cells damaged by ABAP at IC50, as shown in Figure 5. With the increase in the *L. casei* 16‐fermented soymilk extract dosage, the survival rates of the ABAP‐damaged HepG_2_ cells increased. When the water or ethanol extracts concentration of *L. casei* 16‐fermented soymilk was 500 μg/ml, the HepG_2_ cells’ survival rates reached to 74.60 ± 1.24% and 72.90 ± 3.69%, respectively, indicating that the ethanol and water extract of the fermented soymilk protect the HepG_2_ cells against ABAP damage, while water extract of the unfermented soymilk did not enhance the HepG_2_ cells’ survival rates.

**Figure 6:**
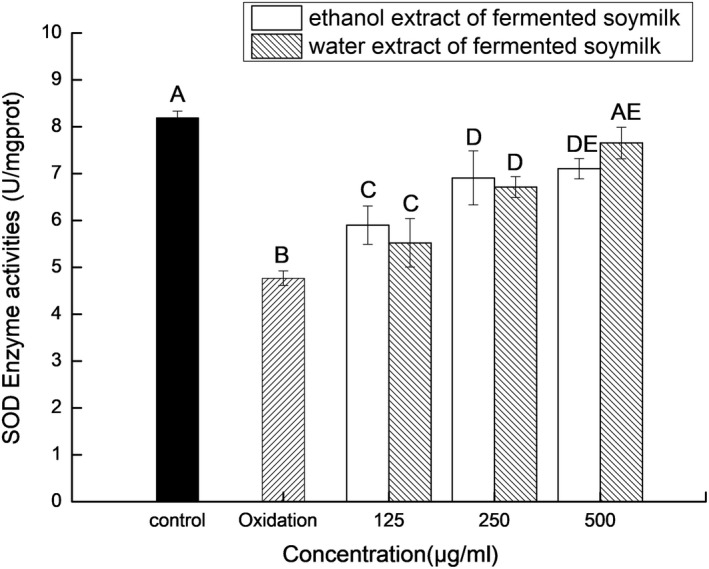
The effect of the extracts of the fermented soymilk on SOD activity of the ABAP‐damaged HepG_2_ cells. Data are represented as mean ± *SD* (*n* = 3). Mean values in the same group with different letters (A‐E) are significantly different by Duncan's multiple range test (*p* < .05)

The SOD activities in HepG_2_ cells treated with ethanol or water extracts of the *L. casei* 16‐fermented soymilk were enhanced as shown in Figure 6. The control group HepG_2_ cells had the highest SOD activities (8.812 ± 0.142 U/mg prot), and the SOD activity of the HepG_2_ cells damaged by ABAP decreased to 4.768 ± 0.153 U/mg prot. When the HepG_2_ cells were pretreated by the *L. casei* 16‐fermented soymilk water or ethanol extracts at the dosage of 125 μg/ml, 250 μg/ml, and 500 μg/ml, respectively, and then damaged by ABAP at IC50, the SOD activities of the HepG_2_ cells were significantly enhanced (*p* < .05) in a dose positive manner. SOD is an enzyme that catalyzes the conversion of superoxide into oxygen and hydrogen peroxide through disproportionation, which protects cells from superoxide damage. Except for reactive oxygen species (ROS) scavenging activity, the *L. casei* 16‐fermented soymilk may enhance SOD activity in the HepG_2_ cells. Li, Long, Pan, Zhao, and Song (2018) reported that *L. plantarum* YS‐1‐fermented soymilk could protect Caco‐2 cells against H_2_O_2_‐induced oxidative damage by reducing the levels of intracellular ROS and enhancing the expression of catalase (CAT), SOD, and glutathione peroxidase (GSH‐Px) in the Caco‐2 cells.

The polyphenols (isoflavones, phenolic acids, and flavanols), free amino acids, and peptides in fermented soybean are responsible for antioxidant activity of the fermented soybean (Sanjukta & Rai, 2016). Yang et al. (2017) reported that the increase of aglycone isoflavones in fermented soymilk explained the antioxidant activity of the fermented soymilk by *L. acidophilus* MF204. Suo et al. (2016) reported that the soymilk fermented by *L. fermentum* Zhao had antioxidant activity due to the higher levels of amino type nitrogen, genistein, and daidzein. In this study, the *L. casei* 16‐fermented soymilk contains higher levels of amino type nitrogen, genistein, and daidzein compared with the unfermented soymilk. Both the water extract and the ethanol extract of the *L. casei* 16‐fermented soymilk could enhance the survival rates and the SOD activities of the ABAP‐damaged HepG_2_ cells. As described above, aglycone isoflavones and total phenolics wer mainly in the ethanol extract, while free amino acids were mainly in the water extract. It can be concluded that the aglycone isoflavones, total phenolics, and free amino acids in the *L. casei* 16‐fermented soymilk were contributed to the protection for HepG_2_ cells against ABAP damage.

### 3.3 Effects of the fermented soymilk extracts on the proliferation of human colon cancer cells

**Figure 7:**
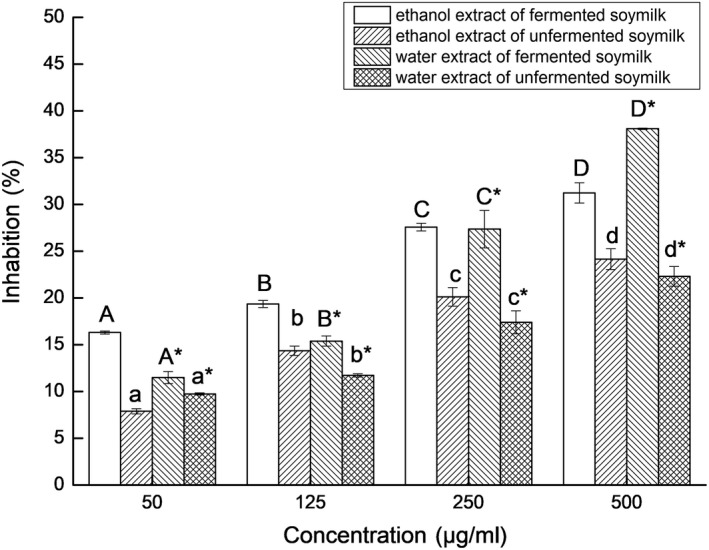
The effects of different concentrations of water (A) and ethanol (B) soymilk extracts on the proliferation of HT‐29. Data are represented as mean ± *SD* (*n* = 3). Mean values in the same group with different letters (a‐d; A‐E; a*‐d*; A*‐D*) are significantly different by Duncan's multiple range test (*p* < .05)

**Figure 8:**
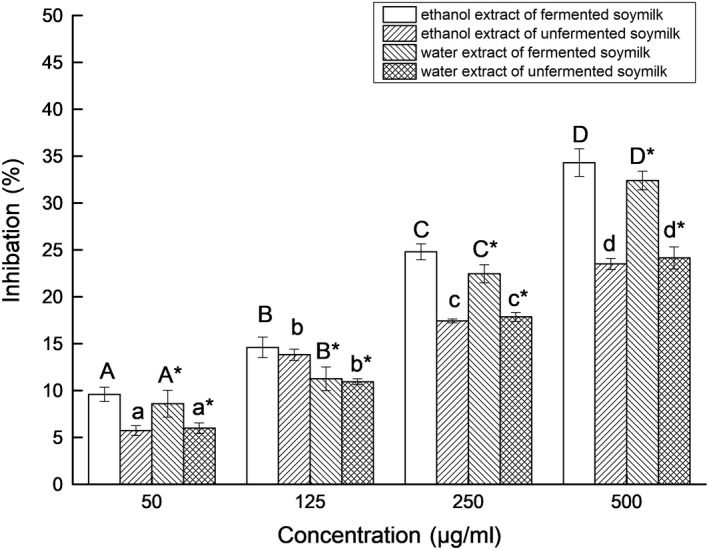
The effects of different concentrations of water and ethanol soymilk extracts on the proliferation of Caco‐2 cells. Data are represented as mean ± *SD* (*n* = 3). Mean values in the same group with different letters (a‐d; A‐E; a*‐d*; A*‐D*) are significantly different by Duncan's multiple range test (*p* < .05)

We further studied the effects of the *L. casei* 16‐fermented soymilk extracts on the proliferation of human colon cancer cell lines, HT‐29 cells, and Caco‐2 cells. As shown in Figures 7 and 8, *L. casei* 16‐fermented soymilk extracts inhibited the proliferation of HT‐29 and Caco‐2 cells in a dose‐dependent manner. Compared with unfermented soymilk, the antiproliferative effect of the *L. casei* 16‐fermented soymilk on HT‐29 and Caco‐2 cells was significantly enhanced (*p* < .05) at the same dose level. This consequence might be due to the increase in the aglycones isoflavones and free amino acids contents in the fermented soymilk as described above.

**Figure 9:**
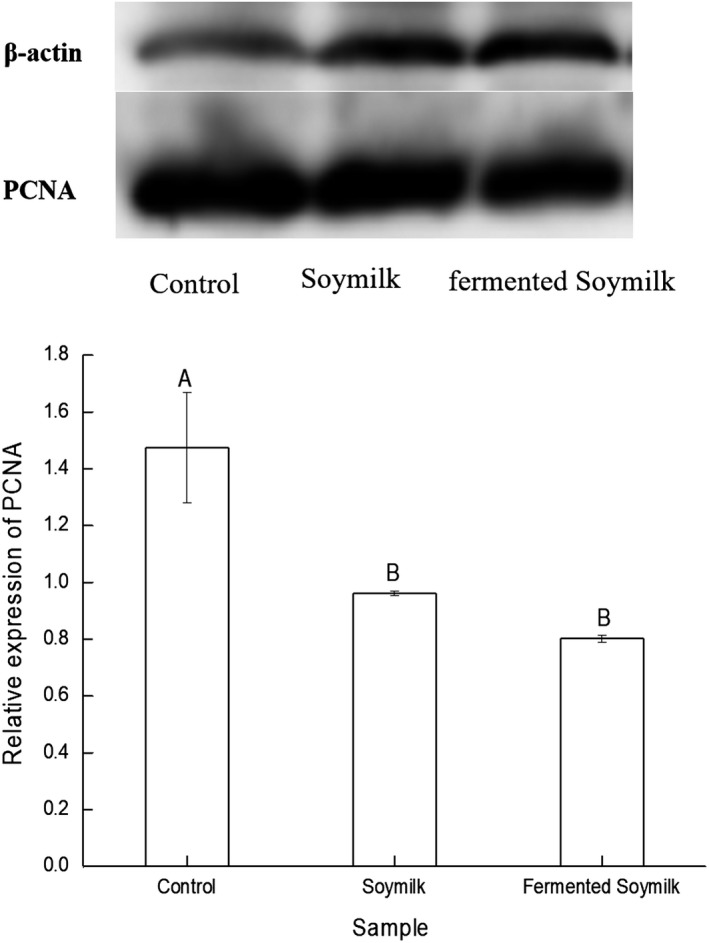
Effects of the extracts of fermented soymilk and nonfermented expression of PCNA in HT‐29 cells. Data are represented as mean ± *SD* (*n* = 3). Mean values in the same group with different letters (A‐B) are significantly different by Duncan's multiple range test (*p* < .05)

To confirm the antiproliferative activity of the *L. casei* 16‐fermented soymilk extracts toward HT‐29 cells, the expression of PCNA in the HT‐29 cells was measured. The expression of PCNA in the HT‐29 cells treated by *L. casei* 16‐fermented soymilk extracts decreased compared with the control HT‐29 cells, but the PCNA expression was not significantly different between the group treated by the extracts of fermented soymilk and other group treated by the extract of nonfermented soymilk, as shown in Figure 9. As shown in Figure 7, both unfermented soymilk and fermented soymilk exerted antiproliferative effect on HT‐29 cells. Lai et al. (2013) reported that soymilk fermented by *S. thermophilus* 14,085 and *B. infantis* 14,603 exerted suppression effect on the proliferation of Caco‐2 and HT‐29 cells, and the ethanol extract exhibited the higher antiproliferative activity. Chen et al. (2013) found that, compared with unfermented soymilk, all the water, 80% methanol or 80% ethanol extract of fermented black soybeans showed significantly higher (*p* < .05) antiproliferative effects on HT‐29 and Caco‐2 cells. Shafiee, Saidijam, Tavilani, Ghasemkhani, and Khodadadi (2016) reported that soybean genistein may exhibit its anticancer properties on HT29 colon cancer cells by modulating caspase‐3 and p38 MAPK pathway at different transcriptional and protein levels. Ye, Li, and Wei (2017) determined that genistein exerted its tumor suppressor effect at least partially via inhibition of S‐phase kinase‐associated protein 2 and promotion of its downstream targets p21 and p27 in breast cancer cells. Khan and Kang (2017) revealed that soybean seed powder fermented with *L. plantarum* DGK‐17 showed antiproliferative effect on human colon cancer HCT‐116 cells through ROS‐JNK signaling pathway. PCNA is often used as a proliferation marker due to its specifically expressed in proliferating cell nuclei. In our study, the ethanol extract and the water extract of the *L. casei* 16‐fermented soymilk showed the suppression effect on the proliferation of both HT‐29 and Caco‐2 cells in a dose manner and inhibited the PCNA expression in HT‐29 cells. In the ethanol extract, the isoflavone aglycone maybe accounts for the antiproliferative effect. In the water extract, the antiproliferative effects may be attributed to the increase in the free amino acid and peptide contents in the fermented soymilk.

## 4 CONCLUSION

This work showed the ethanol and water extracts of the *L. casei* 16‐fermented soymilk could protect HepG_2_ cells against ABAP damage and inhibit the proliferation of human colon cancer cell lines, HT‐29 cells, and Caco‐2 cells. The fermentation of soymilk by *L. casei* 16 enhanced the level of total phenolic, aglycone isoflavones, and free amino acids in the fermented soymilk, which maybe accounts for the protection for the HepG_2_ cells, and the antiproliferative effects on the HT‐29 cells and Caco‐2 cells.

## CONFLICT OF INTEREST

The authors do not have any conflicting interests.

## ETHICAL APPROVAL

This study does not involve any human or animal testing.

## References

[fsn31214-bib-0001] Chen, Y. F. , Chiang, M. L. , Chou, C. C. , & Lo, Y. C. (2013). Enhancing the antitumor cell proliferation and Cu 2+ ‐chelating effects of black soybeans through fermentation with *Aspergillus awamori* . Journal of Bioscience & Bioengineering, 115(4), 400–404. 10.1016/j.jbiosc.2012.10.018 23177213

[fsn31214-bib-0002] Dajanta, K. , Chukeatirote, E. , & Apichartsrangkoon, A. (2012). Improvement of thua nao production using protein‐rich soybean and Bacillus subtilis TN51 starter culture. Annals of Microbiology, 62(2), 785–795. 10.1007/s13213-011-0319-1

[fsn31214-bib-0003] Dajanta, K. , Janpum, P. , & Leksing, W. (2013). Antioxidant capacities, total phenolics and flavonoids in black and yellow soybeans fermented by *Bacillus subtilis*: A comparative study of Thai fermented soybeans (thua nao). International Food Research Journal, 20, 3125–3132.

[fsn31214-bib-0004] Dini, I. , Schettino, O. , Simioli, T. , & Dini, A. (2001). Studies on the constituents of Chenopodium quinoa seeds: Isolation and characterization of new triterpene saponins. Journal of Agricultural & Food Chemistry, 49(2), 741. 11262022 10.1021/jf000971y

[fsn31214-bib-0005] Donkor, O. N. , & Shah, N. P. (2010). Production of beta‐glucosidase and hydrolysis of isoflavone phytoestrogens by Lactobacillus acidophilus, *Bifidobacterium lactis*, and *Lactobacillus casei* in soymilk. Journal of Food Science, 73(1), M15–M20. 10.1111/j.1750-3841.2007.00547.x 18211356

[fsn31214-bib-0006] Erdman, J. W. , & Committee, F. N. (2000). Soy protein and cardiovascular disease a statement for healthcare professionals from the Nutrition Committee of the AHA. Circulation, 102(20), 2555–2559 11076833 10.1161/01.cir.102.20.2555

[fsn31214-bib-0007] Guan, Y. , Wang, J. , Wu, J. , Wang, L. , Rui, X. , Xing, G. , & Dong, M. (2017). Enhancing the functional properties of soymilk residues (okara) by solid‐state fermentation with Actinomucor elegans. CyTA – Journal of Food, 15(1), 155–163.

[fsn31214-bib-0008] Han, S. S. , Hur, S. J. , & Lee, S. K. (2015). A comparison of antioxidative and anti‐inflammatory activities of sword beans and soybeans fermented with *Bacillus subtilis* . Food & Function, 6(8), 2736–2748. 10.1039/C5FO00290G 26149963

[fsn31214-bib-0009] Hu, Y. J. , Ge, C. R. , Wei, Y. , Zhu, R. J. , Zhang, W. J. , Du, L. J. , & Jie, X. (2010). Characterization of fermented black soybean natto inoculated with Bacillus natto during fermentation. Journal of the Science of Food & Agriculture, 90(7), 1194–1202. 20394001 10.1002/jsfa.3947

[fsn31214-bib-0010] Jane, H. , Monique, B. , FrançOise, N. , FrançOis, P. , & Jean, D. (2008). Effects of fermentation on the phytochemical composition and antioxidant properties of soy germ. Food Chemistry, 109(4), 709–721. 10.1016/j.foodchem.2007.12.081 26049983

[fsn31214-bib-0011] Jiyeon, C. , Gyoung Min, K. , Wook, L. K. , In Duck, C. , Gun‐Hee, K. , Jae‐Young, P. , … Jeong Hwan, K. (2010). Conversion of isoflavone glucosides to aglycones in soymilk by fermentation with lactic acid bacteria. Journal of Food Science, 72(2), M39–M44. 10.1111/j.1750-3841.2007.00276.x 17995840

[fsn31214-bib-0012] Khan, I. , & Kang, S. C. (2017). Apoptotic activity of *Lactobacillus plantarum* DGK‐17‐fermented soybean seed extract in human colon cancer cells via ROS‐JNK signaling pathway. Journal of Food Science, 82(4):1475‐1483. 28488794 10.1111/1750-3841.13732

[fsn31214-bib-0013] Lai, L. R. , Hsieh, S. C. , Huang, H. Y. , & Chou, C. C. (2013). Effect of lactic fermentation on the total phenolic, saponin and phytic acid contents as well as anti‐colon cancer cell proliferation activity of soymilk. Journal of Bioscience & Bioengineering, 115(5), 552–556. 10.1016/j.jbiosc.2012.11.022 23290992

[fsn31214-bib-0014] Landete, J. M. , Curiel, J. A. , Rodríguez, H. , Rivas, B. D. L. , & Muñoz, R. (2014). Aryl glycosidases from Lactobacillus plantarum increase antioxidant activity of phenolic compounds. Journal of Functional Foods, 7(1), 322–329. 10.1016/j.jff.2014.01.028

[fsn31214-bib-0015] Lee, I.‐H. , Hung, Y.‐H. , & Chou, C.‐C. (2008). Solid‐state fermentation with fungi to enhance the antioxidative activity, total phenolic and anthocyanin contents of black bean. International Journal of Food Microbiology, 121(2), 150–156. 10.1016/j.ijfoodmicro.2007.09.008 18031859

[fsn31214-bib-0016] Li, G. , Long, X. , Pan, Y. , Zhao, X. , & Song, J.‐L. (2018). Study on soybean milk fermented by Lactobacillus plantarum YS‐1 reduced the H2O2‐induced oxidative damage in Caco‐2 cells. Biomedical Research, 29(2), 357–364. 10.4066/biomedicalresearch.29-17-3132

[fsn31214-bib-0017] Marazza, J. A. , Garro, M. S. , & de Giori, G. S. (2009). Aglycone production by *Lactobacillus rhamnosus* CRL981 during soymilk fermentation. Food Microbiology, 26(3), 333–339. 10.1016/j.fm.2008.11.004 19269578

[fsn31214-bib-0018] Marazza, J. A. , Nazareno, M. A. , Giori, G. S. D. , & Garro, M. S. (2012). Enhancement of the antioxidant capacity of soymilk by fermentation with *Lactobacillus rhamnosus* . Journal of Functional Foods, 4(3), 594–601. 10.1016/j.jff.2012.03.005

[fsn31214-bib-0019] Ming‐Yen, J. , & Cheng‐Chun, C. (2010). Enhancement of antioxidant activity, total phenolic and flavonoid content of black soybeans by solid state fermentation with Bacillus subtilis BCRC 14715. Food Microbiology, 27(5), 586–591. 10.1016/j.fm.2009.11.002 20510775

[fsn31214-bib-0020] Moktan, B. , Saha, J. , & Sarkar, P. K. (2008). Antioxidant activities of soybean as affected by Bacillus‐fermentation to kinema. Food Research International, 41(6), 586–593. 10.1016/j.foodres.2008.04.003

[fsn31214-bib-0021] Mu, G. , Gao, Y. , Tuo, Y. , Li, H. , Zhang, Y. , Qian, F. , & Jiang, S. (2018). Assessing and comparing antioxidant activities of lactobacilli strains by using different chemical and cellular antioxidant methods. Journal of Dairy Science, 101, 10792‐10806. 30268622 10.3168/jds.2018-14989

[fsn31214-bib-0022] Ohta, T. , Nakatsugi, S. , Watanabe, K. , Kawamori, T. , Ishikawa, F. , Morotomi, M. , … Wakabayashi, K. (2000). Inhibitory effects of Bifidobacterium‐fermented soy milk on 2‐amino‐1‐methyl‐6‐phenylimidazo[4,5‐b]pyridine‐induced rat mammary carcinogenesis, with a partial contribution of its component isoflavones. Carcinogenesis, 21(5), 937–941. 10.1093/carcin/21.5.937 10783315

[fsn31214-bib-0023] Pham, T. T. , & Shah, N. P. (2008). Effects of lactulose supplementation on the growth of bifidobacteria and biotransformation of isoflavone glycosides to isoflavone aglycones in soymilk. Journal of Agricultural & Food Chemistry, 56(12), 4703 10.1021/jf072716k 18500812

[fsn31214-bib-0024] Rekha, C. R. , & Vijayalakshmi, G. (2011). Isoflavone phytoestrogens in soymilk fermented with β‐glucosidase producing probiotic lactic acid bacteria. International Journal of Food Sciences & Nutrition, 62(2), 111–120. 10.3109/09637486.2010.513680 21091296

[fsn31214-bib-0025] Rui, X. , Wang, M. , Zhang, Y. , Chen, X. , Li, L. , Liu, Y. , & Dong, M. (2017). Optimization of soy solid‐state fermentation with selected lactic acid bacteria and the effect on the anti‐nutritional components. Journal of Food Processing & Preservation, 41(6), e13290 10.1111/jfpp.13290

[fsn31214-bib-0026] Sanjukta, S. , & Rai, A. K. (2016). Production of bioactive peptides during soybean fermentation and their potential health benefits. Trends in Food Science & Technology, 50, 1–10. 10.1016/j.tifs.2016.01.010

[fsn31214-bib-0027] Sanjukta, S. , Rai, A. K. , Muhammed, A. , Jeyaram, K. , & Talukdar, N. C. (2015). Enhancement of antioxidant properties of two soybean varieties of Sikkim Himalayan region by proteolytic Bacillus subtilis fermentation. Journal of Functional Foods, 14, 650–658. 10.1016/j.jff.2015.02.033

[fsn31214-bib-0028] Shafiee, G. , Saidijam, M. , Tavilani, H. , Ghasemkhani, N. , & Khodadadi, I. (2016). Genistein Induces Apoptosis and Inhibits Proliferation of HT29 Colon Cancer Cells. International Journal of Molecular & Cellular Medicine, 5(3), 178–191. 27942504 PMC5125370

[fsn31214-bib-0029] Song, W. , Derito, C. M. , Liu, M. K. , He, X. , Dong, M. , & Liu, R. H. (2010). Cellular antioxidant activity of common vegetables. Journal of Agricultural and Food Chemistry, 58(11), 6621–6629. 10.1021/jf9035832 20462192

[fsn31214-bib-0030] Su, L. , Cheng, Y. , Hsiao, F. , Han, J. , & Yu, Y. (2018). Optimization of Mixed Solid-state Fermentation of Soybean Meal by Lactobacillus Species and Clostridium butyricum. Polish Journal of Microbiology, 67(3), 297–305. 30451446 10.21307/pjm-2018-035 PMC7255691

[fsn31214-bib-0031] Suo, H. , Yu, Q. , Xia, F. , Wang, H. , Xin, Z. , & Song, J. L. (2016). Free radical scavenging activity and cytoprotective effect of soybean milk fermented with Lactobacillus Fermentum Zhao. Journal of Food Biochemistry, 40(3), 294–303.

[fsn31214-bib-0032] Tang, Y. , Zhang, J. , Gao, Y. , Li, H. , Zhang, Y. , & Tuo, Y. (2018). Study on Antioxidant Properties of Fermented Soymilk by Lactobacillus casei-16. Food Research and Development, 39(8), 1–9.

[fsn31214-bib-0033] Watanabe, N. , Fujimoto, K. , & Aoki, H. (2007). Antioxidant activities of the water‐soluble fraction in tempeh‐like fermented soybean (GABA‐tempeh). International Journal of Food Sciences & Nutrition, 58(8), 577–587. 10.1080/09637480701343846 17852485

[fsn31214-bib-0034] Wei, Q. K. , Chen, T. R. , & Chen, J. T. (2007). Using of Lactobacillus and Bifidobacterium to product the isoflavone aglycones in fermented soymilk. International Journal of Food Microbiology, 117(1), 120–124. 10.1016/j.ijfoodmicro.2007.02.024 17477997

[fsn31214-bib-0035] Wen‐Huei, C. , Jun‐Jen, L. , Ching‐Hsein, C. , Tien‐Shang, H. , & Fung‐Jou, L. (2002). Growth inhibition and induction of apoptosis in MCF‐7 breast cancer cells by fermented soy milk. Nutrition & Cancer, 43(2), 13. 10.1207/S15327914NC432_12 12588701

[fsn31214-bib-0036] Wolfe, K. L. , & Hai, L. R. (2008). Structure‐activity relationships of flavonoids in the cellular antioxidant activity assay. Journal of Agriculture and Food Chemistry, 56(18), 8404–8411. 10.1021/jf8013074 18702468

[fsn31214-bib-0037] Xing, G. , Rui, X. , Wang, D. , Liu, M. , Chen, X. , & Dong, M. (2017). The effect of fermentation pH on the protein bioaccessibility of soymilk curd with added tea polyphenols as assessed by in vitro gastrointestinal digestion. Journal of Agricultural & Food Chemistry, 65(50), 11125–11132. 29185340 10.1021/acs.jafc.7b04456

[fsn31214-bib-0038] Yan, S. , Wei, P. C. , Chen, Q. , Chen, X. , Wang, S. C. , Li, J. R. , & Gao, C. (2018). Functional and structural characterization of a β‐glucosidase involved in saponin metabolism from intestinal bacteria. Biochemical & Biophysical Research Communications, 496(4), 1349–1356. 10.1016/j.bbrc.2018.02.018 29421652

[fsn31214-bib-0039] Yang, C. , Yan, W. , Jie, C. , Xu, X. , Meng, Y. , Yang, C. , Meng, Y. (2017). Antioxidant and hypolipidemic effects of soymilk fermented via Lactococcus acidophilus MF204. Food & Function, 8(12):4414–4420. 29085943 10.1039/c7fo00701a

[fsn31214-bib-0040] Ye, D. , Li, Z. , & Wei, C. (2017). Genistein inhibits the S‐phase kinase‐associated protein 2 expression in breast cancer cells. Experimental & Therapeutic Medicine, 15(1), 1069–1075. 10.3892/etm.2017.5489 29434697 PMC5772955

[fsn31214-bib-0041] Zhai, Q. , Xiao, Y. , Tian, F. , Wang, G. , Zhao, J. , Liu, X. , … Chen, W. (2014). Protective effects of lactic acid bacteria‐fermented soymilk against chronic cadmium toxicity in mice. RSC Advances, 5(6), 4648–4658. 10.1039/C4RA12865F

[fsn31214-bib-0042] Zhang, J. H. , Tatsumi, E. , Fan, J. F. , & Li, L. T. (2010). Chemical components of Aspergillus ‐type Douchi, a Chinese traditional fermented soybean product, change during the fermentation process. International Journal of Food Science & Technology, 42(3), 263–268. 10.1111/j.1365-2621.2005.01150.x

